# THE INCORPORATING NUTRITION, VESTS, EDUCATION, AND STRENGTH TRAINING IN BONE HEALTH RANDOMIZED TRIAL RESULTS

**DOI:** 10.1093/geroni/igae098.1053

**Published:** 2024-12-31

**Authors:** Kristen Beavers, Daniel Beavers, Jason Fanning, Leon Lenchik, Barbara Nicklas, Ashley Weaver

**Affiliations:** Wake Forest University School of Medicine, Winston-Salem, North Carolina, United States; Wake Forest University, Winston-Salem, North Carolina, United States; Wake Forest University, Winston-Salem, North Carolina, United States; Wake Forest University School of Medicine, Winston-Salem, North Carolina, United States; Wake Forest University School of Medicine, Winston-Salem, North Carolina, United States; Wake Forest University School of Medicine, Winston-Salem, North Carolina, United States

## Abstract

Intentional weight loss (WL) in older adults is associated with bone loss, increasing risk of fracture. As skeletal tissue is responsive to mechanical stress, replacing lost weight externally may be an innovative way to minimize WL-associated bone loss in this population. The main goal of the Incorporating Nutrition, Vests, Education, and Strength Training (INVEST) in Bone health trial (NCT04076618) is to examine the effect of 12-months of weighted vest use during intentional WL on several indicators of bone health, as compared to WL alone and WL plus traditional strength training exercise. Herein we report intervention process measure data, including weight change (%), self-reported weighted vest wear-time (hours/day), lost weight added to the weighted vest (%), and strength training adherence (% sessions attended; days/week). A total of 150 older adults were randomized, with 133 (89%) completing the trial. Average age was 66.4±4.6 years and the majority were white (69%) women (75%) with a body mass index of 33.6±3.3 kg/m2. 12-month WL by group was 9.4±6.7%, 9.0±4.7%, 11.3±6.0% for WL alone, WL+weighted vest, and WL+strength training, respectively. Self-reported weighted vest wear-time over 12-months was 7.1±1.5 hours/day, with 81.8±22.8% of lost weight replaced in the vest. Participants assigned to WL+strength training attended 75.2±13.0% of classes, averaging 2.2±0.4 sessions attended/week over 12-months. Intervention process measure data from the INVEST in Bone Health trial suggest significant and similar WL in each group, with good adherence to the weighted vest and exercise prescriptions. This presentation will also show treatment effects on select measures of musculoskeletal health.

